# TLR2/MyD88/NF-κB Pathway, Reactive Oxygen Species, Potassium Efflux Activates NLRP3/ASC Inflammasome during Respiratory Syncytial Virus Infection

**DOI:** 10.1371/journal.pone.0029695

**Published:** 2012-01-25

**Authors:** Jesus Segovia, Ahmed Sabbah, Victoria Mgbemena, Su-Yu Tsai, Te-Hung Chang, Michael T. Berton, Ian R. Morris, Irving C. Allen, Jenny P.-Y. Ting, Santanu Bose

**Affiliations:** 1 Department of Microbiology and Immunology, The University of Texas Health Science Center at San Antonio, San Antonio, Texas, United States of America; 2 Department of Microbiology-Immunology, Lineberger Comprehensive Cancer Center, University of North Carolina, Chapel Hill, North Carolina, United States of America; University of California Merced, United States of America

## Abstract

Human respiratory syncytial virus (RSV) constitute highly pathogenic virus that cause severe respiratory diseases in newborn, children, elderly and immuno-compromised individuals. Airway inflammation is a critical regulator of disease outcome in RSV infected hosts. Although “controlled” inflammation is required for virus clearance, aberrant and exaggerated inflammation during RSV infection results in development of inflammatory diseases like pneumonia and bronchiolitis. Interleukin-1β (IL-1β) plays an important role in inflammation by orchestrating the pro-inflammatory response. IL-1β is synthesized as an immature pro-IL-1β form. It is cleaved by activated caspase-1 to yield mature IL-1β that is secreted extracellularly. Activation of caspase-1 is mediated by a multi-protein complex known as the inflammasome. Although RSV infection results in IL-1β release, the mechanism is unknown. Here in, we have characterized the mechanism of IL-1β secretion following RSV infection. Our study revealed that NLRP3/ASC inflammasome activation is crucial for IL-1β production during RSV infection. Further studies illustrated that prior to inflammasome formation; the “first signal” constitutes activation of toll-like receptor-2 (TLR2)/MyD88/NF-κB pathway. TLR2/MyD88/NF-κB signaling is required for pro-IL-1β and NLRP3 gene expression during RSV infection. Following expression of these genes, two “second signals” are essential for triggering inflammasome activation. Intracellular reactive oxygen species (ROS) and potassium (K^+^) efflux due to stimulation of ATP-sensitive ion channel promote inflammasome activation following RSV infection. Thus, our studies have underscored the requirement of TLR2/MyD88/NF-κB pathway (first signal) and ROS/potassium efflux (second signal) for NLRP3/ASC inflammasome formation, leading to caspase-1 activation and subsequent IL-1β release during RSV infection.

## Introduction

Human respiratory syncytial virus (RSV) is a RNA respiratory virus that infects lung epithelial cells to cause high mortality and morbidity among infants, children and elderly by developing severe respiratory diseases like pneumonia and bronchiolitis [1]–[3]. These diseases occur due to massive and “uncontrolled” inflammation of the respiratory tract. It is believed that prolonged virus infection (and resulting high viral replication/multiplication) results in a robust inflammatory response that is detrimental to the infected host. The innate immune antiviral response is the first line of defense against virus infection before induction of the adaptive immune response [4]–[6]. It is well established that innate response is critical to restrict virus spread and infection resulting in diminished disease burden. The inflammatory response constitutes a critical innate defense mechanism triggered by the host to control infections [7]. However, aberrant and unregulated inflammation results in development of various disease states including pneumonia and bronchiolitis.

Interleukin-1β (IL-1β) is a critical cytokine that acts as a pyrogen to amplify the pro-inflammatory response during infection with various pathogens. IL-1β produced from infected cells acts via an autocrine/paracrine mechanism to activate NF-κB/MAP kinase dependent pro-inflammatory cytokines and chemokines to establish an effective immune response for combating infection. Respiratory RNA viruses like RSV and influenza A virus induce secretion of IL-1β in the respiratory tract during infection of mouse and humans and its secretion is critical for “shaping” the anti-viral inflammatory response to clear virus from the airway [8]–[12]. Production of IL-1β from macrophages requires three steps – a) expression of pro-IL-1β gene and synthesis of immature pro-IL-1β protein, b) processing (cleavage) of pro-IL-1β by active caspase-1 to yield the mature form of IL-1β, and c) secretion of mature IL-1β from the cell to the extracellular environment via Rab-3a containing secretory vesicle [13]. Generation of mature IL-1β is achieved following cytoplasmic assembly and activation of inflammasomes [14]–[19]. The NLR (nucleotide binding oligomerization domain like receptor) inflammasome is a multi-protein complex comprising of caspase-1, NLR proteins and adaptor protein ASC (apoptosis-associated speck-like protein containing a caspase recruitment domain) [20]–[22]. The well characterized NLR inflammasome complex constitutes NLR protein NLRP3 (NOD-like receptor family, pryin domain containing 3; also known as NALP3 and cryopyrin), ASC and caspase-1. Oligomerization of NALP3-ASC results in caspase-1 recruitment in the complex, which promotes cleavage of casapase-1 via auto-catalytic mechanism to generate enzymatically active hetero-dimer of two p20 and p10 subunits, which are involved in cleaving the precursor pro-IL-1β into its mature secreted form [14]–[22]. Various stress signals culminates in formation of inflammasome complex during diverse patho-physiological conditions/disease like infection, cardiovascular disease, metabolic disorder (e.g. diabetes), and inflammatory diseases (arthritis, gout) [14]–[37].

During stress response, macrophages need two signals for inflammasome complex formation and subsequent caspase-1 activation [38]. First signal (signal-1) ensures adequate gene/protein expression of pro-IL-1β and inflammasome components (e.g. NLRP3 etc); while second signal (signal-2) is required for inflammasome complex assembly and subsequent caspase-1 activation leading to cleavage of pro-IL-1β into its mature form. Following cellular insult, signal-1 is mediated by stimulation of pattern recognition receptors (PRRs) (e.g. toll-like receptors or TLRs, NOD-like receptors like Nod2) via Pathogen Associated Molecular Patterns (PAMPs) or Damage Associated Molecular Patterns (DAMPs) which are specific “molecular” or “chemical” signature associated with the cellular stimuli [21], [22], [39]. PRR induction results in activation of NF-κB pathway for expression of pro-IL-1β and inflammasome related protein genes. While signal-2 is conferred by either – a) generation of intracellular reactive oxygen species (ROS), b) potassium efflux due to stimulation of ATP-sensitive potassium channel or pore formation by bacterial toxins, and c) lysosomal disintegration leading to leakage of cathepsin B in the cytosol [14]–[19], [29].

Although RSV infection results in IL-1β secretion [9]–[12], the mechanism of IL-1β activation has not been elucidated yet. Recently we identified Nod2 (a member of NLR family of receptors) as a competent of innate antiviral response against RSV [40]. Since NLRs like NLRP3 is a component of inflammasome complex [14]–[37], we investigated – a) the requirement of activated caspase-1 for IL-1β secretion during RSV infection, b) the nature of NLR inflammasome complex assembled following RSV infection, c) the role of PRRs and NF-κB pathway in expression of pro-IL-1β and inflammasome related genes during RSV infection (signal-1), and d) the specific mechanism responsible for triggering activation of inflammasome complex (signal-2). Our study revealed that during RSV infection NLRP3-ASC inflammasome is required for caspase-1 activation and mature IL-1β secretion. RSV mediated activation of TLR2/MyD88 signaling is critical for expression of pro-IL-1β and NLRP3 genes via NF-κB dependent pathway (signal-1). Both ROS generated during RSV infection and potassium efflux following stimulation of ATP-sensitive potassium channel serve as the second signal (signal-2) for inflammasome mediated generation of mature IL-1β due to cleavage of pro-IL-1β by activated caspase-1. Thus our results have underscored the requirement of TLR2-MyD88-NF-κB pathway and NLRP3-ASC inflammasome in IL-1β release during RSV infection. Furthermore, we have identified both ROS and potassium efflux (due to stimulation of potassium channel) as the intracellular signal essential for triggering inflammasome activation and IL-1β production during infection.

## Results

### Caspase-1 is required for IL-1β production during RSV infection

**Figure 1:**
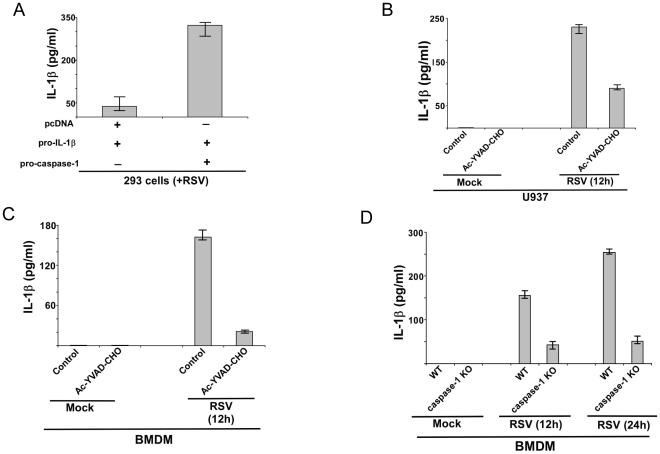
Caspase-1 dependent IL-1β release during RSV infection. (**A**) 293 cells were transfected either with pcDNA (control), pro-IL-1β and/or pro-caspase-1 plasmids. At 24 h post-transfection, cells were infected with RSV (1 MOI). At 12 h post-infection, medium supernatant was assessed for IL-1β protein by ELISA analysis. (**B**) Human macrophagic U937 cells were infected with RSV (1 MOI) in the presence of either water (vehicle control) or caspase-1 inhibitor (10 µM of Ac-YVAD-CHO). IL-1β levels in the medium supernatant were assayed by ELISA at 12 h post-infection. (**C**) Primary mouse bone marrow derived macrophages (BMDM) were infected with RSV (1 MOI) in the presence of either water (vehicle control) or caspase-1 inhibitor (10 µM of Ac-YVAD-CHO). IL-1β levels in the medium supernatant were assayed by ELISA at 12 h post-infection. (**D**) Wild type (WT) or caspase-1 knock-out (KO) BMDMs were infected with RSV (1 MOI). IL-1β levels in the medium supernatant were assayed by ELISA at 12 h and 24 h post-infection. Each value represents the mean ± standard deviation from three independent experiments.

The role of caspase-1 in IL-1β secretion during RSV infection was examined by over-expressing pro-caspase-1 in 293 cells (obtained from American Type Culture Collection (ATCC), Manassas, VA) that endogenously express low levels of pro-caspase-1. 293 cells were transfected with pro-IL-1β, pro-caspase-1 or pcDNA (control) plasmids. Twenty four hour post-transfection, cells were infected with RSV. At 12 h post-infection, the medium supernatant was collected to assess levels of secreted IL-1β by ELISA. Expression of pro-IL-1β and pro-caspase-1 in transfected 293 cells was confirmed by RT-PCR (data not shown). Pro-IL-1β expression was similar in pro-IL-1β+pcDNA transfected cells and pro-IL-1β+pro-caspase-1 transfected 293 cells (data not shown). Caspase-1 was required for IL-1β production during RSV infection, since higher levels of secreted IL-1β was detected in pro-caspase-1 expressing cells (i.e. pro-caspase-1+pro-IL-1β) compared to control cells (i.e. cells transfected with pcDNA+pro-IL-1β) (Fig. 1A). There was an enhancement of IL-1β production by 300 pg/ml following expression of caspase-1 in 293 cells (Fig. 1A). Low levels of basal IL-1β release in pro-caspase-1 non-transfected cells (Fig. 1A) could be due to activation of endogenous pro-caspase-1 in 293 cells. However, we failed to detect caspase-1 in mock infected and RSV infected 293 cells (Fig. S1A). Similarly, pro-IL-1β was not expressed in 293 cells (Fig. S1B). Caspase-1 (Fig. S1A) and pro-IL-1β (Fig. S1B) expression was only detected following transfection of pro-caspase-1 and pro-IL-1β. Thus, basal IL-1β production from 293 cells could be due to an alternative mechanism, which is yet to be characterized. Moreover, non-infected (mock) cells transfected with pro-IL-1β and pro-caspase-1 secreted very low levels (less than 10 pg/ml) of IL-1β (data not shown), which could be attributed to either self activation of caspase-1 or activation of alternate discrete signaling in 293 cells.

To establish the physiological role of caspase-1, we next evaluated the requirement of endogenous caspase-1 in IL-1β production. For these studies we utilized human macrophage-like U937 cell-line (obtained from American Type Culture Collection (ATCC), Manassas, VA), since macrophages constitutes the major source of IL-1β during respiratory virus infection. Inhibition of caspase-1 by specific caspase-1 inhibitor Ac-YVAD-CHO [41] resulted in drastic reduction in IL-1β secretion following RSV infection for 12 h (Fig. 1B). These studies were further extended to primary macrophages isolated from mice. Similar to U937 cells, treatment of mouse bone marrow derived macrophages (BMDM) with caspase-1 inhibitor Ac-YVAD-CHO resulted is loss of IL-1β production from RSV infected cells (Fig. 1C). The release of IL-1β from RSV infected U937 and BMDMs at 12 h–24 h post-infection was not due to cell death (apoptosis, pyroptosis or necrosis) because annexin-V and Propidium Iodide (PI) staining (representative of cell death) was not observed in RSV infected (at 12 h–24 h post-infection) U937 cells and BMDMs (data not shown). Annexin-V staining was noted only at 48 h post-infection. Interestingly, we failed to detect IL-1β secretion from RSV infected human epithelial cell-lines [human lung epithelial A549 (obtained from American Type Culture Collection (ATCC), Manassas, VA) cells and Hela (obtained from American Type Culture Collection (ATCC), Manassas, VA) cells (data not shown). In contrast, normal human bronchial epithelial (NHBE) primary cells (obtained from Lonza, Walkersville, MD) which are one of the primary targets of RSV during productive infection of human lungs produced IL-1β following RSV infection (Fig. S2). The IL-1β release was due to caspase-1 activation, since inhibition of caspase-1 activity by caspase-1 inhibitor Ac-YVAD-CHO drastically reduced IL-1β secretion from RSV infected NHBE cells (Fig. S2).

The result obtained with caspase-1 inhibitor was further validated by using primary cells that lack caspase-1 expression. Wild-type (WT) and caspase-1 knock-out (KO) BMDMs were infected with RSV for 12 h–24 h. At each time-period, IL-1β secretion was assayed by ELISA analysis of medium supernatants. Caspase-1 expression is vital for IL-1β release, since levels of IL-1β was drastically diminished in the medium supernatant of infected caspase-1 KO cells compared to WT cells (Fig. 1D). Thus our results obtained with macrophage cell-line and primary macrophages demonstrated that caspase-1 plays a critical role in mature IL-1β processing and secretion during RSV infection.

### NLRP3 inflammasome is required for IL-1β release during RSV infection

**Figure 2:**
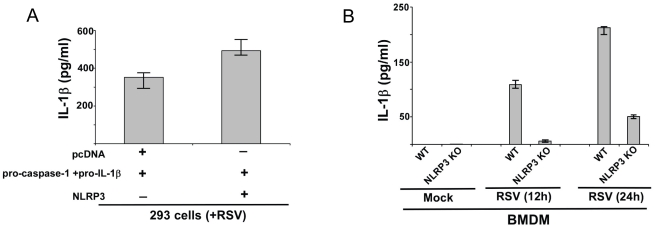
IL-1β secretion during RSV infection requires NLRP3 expression. (**A**) 293 cells were transfected either with pcDNA (control), pro-IL-1β+pro-caspase-1 plasmids and NLRP3 plasmid. At 24 h post-transfection, cells were infected with RSV (1 MOI). At 12 h post-infection, medium supernatant was assessed for IL-1β protein by ELISA analysis. (**B**) Wild type (WT) or NLRP3 knock-out (KO) BMDMs were infected with RSV (1 MOI). IL-1β levels in the medium supernatant were assayed by ELISA at 12 h and 24 h post-infection. Each value represents the mean ± standard deviation from three independent experiments.

Caspase-1 activation is mediated by inflammasome complex. Since IL-1β production during RSV infection was caspase-1 dependent (Fig. 1), we speculated that NLR protein(s) is required for caspase-1 activation following inflammasome assembly. We first focused on the NLRP3 since this NLR protein is an important component of inflammasome complex during infections with various pathogens, including respiratory viruses like influenza A virus [30]–[32]. The role of NLRP3 was evaluated by investigating IL-1β production from RSV infected 293 cells expressing NLRP3 in trans. 293 cells transfected with pro-IL-1β, pro-caspase-1 and NLRP3 plasmids were infected with RSV. IL-1β production from these cells was examined by performing ELISA analysis of the medium supernatants. NLRP3 plays a role during IL-1β production, since enhanced IL-1β levels were detected in infected NLRP3 expressing cells (i.e. NLRP3+pro-caspase-1+pro-IL-1β) compared to cells transfected with pcDNA (control) (pcDNA+pro-caspase-1+pro-IL-1β) (Fig. 2A). There was an enhancement of IL-1β production by 150 pg/ml following expression of NLRP3 in 293 cells (Fig. 2A). Expression of pro-IL-1β, caspase-1 and NLRP3 in transfected 293 cells was confirmed by RT-PCR (data not shown). Pro-IL-1β and caspase-1 expression was similar in pro-IL-1β+pro-caspase-1+pcDNA transfected cells and pro-IL-1β+pro-caspase-1+NLRP3 transfected 293 cells (data not shown). It is important to mention that RSV infection induced endogenous NLRP3 expression in 293 cells (Fig. S1C), thus over-expression (following transfection with NLRP3 plasmid as shown in Fig. S1C) of NLRP3 stimulated increased IL-1β production from RSV infected 293 cells.

The role of NLRP3 during IL-1β secretion was further validated using mouse primary BMDMs lacking NLRP3 expression (NLRP3 KO BMDM). WT and NLRP3 KO BMDMs were infected with RSV and at 12 h and 24 h post-infection time periods medium supernatant was collected to assess IL-1β levels by ELISA. As shown in Fig. 2B, NLRP3 expression is critical for IL-1β secretion since drastic reduction in IL-1β release was observed in RSV infected NLRP3 KO cells compared to WT cells. These results demonstrated that NLRP3 is an important component of the inflammasome complex and therefore, it is required for optimal caspase-1 activation and IL-1β secretion from RSV infected cells.

### ASC is required for IL-1β secretion during RSV infection

**Figure 3:**
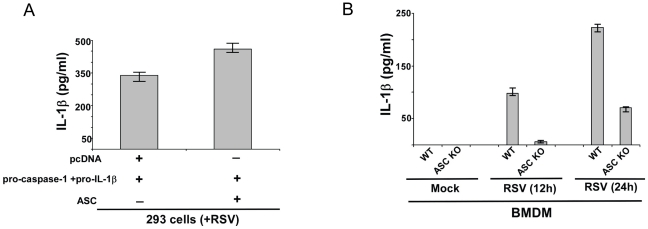
ASC expression is essential for IL-1β production during RSV infection. (**A**) 293 cells were transfected either with pcDNA (control), pro-IL-1β+pro-caspase-1 plasmids and ASC plasmid. At 24 h post-transfection, cells were infected with RSV (1 MOI). At 12 h post-infection, medium supernatant was assessed for IL-1β protein by ELISA analysis. (**B**) Wild type (WT) or ASC knock-out (KO) BMDMs were infected with RSV (1 MOI). IL-1β levels in the medium supernatant were assayed by ELISA at 12 h and 24 h post-infection. Each value represents the mean ± standard deviation from three independent experiments.

Dissection of the constituents of the inflammasome complex has revealed that ASC protein serves as a major component of NLRP3 inflammasome. NLRP3/ASC inflammasome complex has been implicated in caspase-1 activation during various physiological and pathological conditions. Since we identified NLRP3 as an important inflammasome complex component during RSV infection (Fig. 2), we next examined whether ASC/NLRP3 inflammasome is required for IL-1β secretion during RSV infection. Similar to above studies with NLRP3 (Fig. 2), we initially evaluated the role of ASC by utilizing pro-IL-1β and pro-caspase-1 expressing 293 cells. These cells were transfected with either ASC or pcDNA (control) plasmids. After 24 h post-transfection, RSV infection was initiated and IL-1β secretion was measured by performing IL-1β ELISA assay with the medium supernatants. ASC also serve as an essential factor regulating IL-1β production, since higher levels of IL-1β was detected in RSV infected ASC expressing (i.e. ASC+pro-caspase-1+pro-IL-1β) 293 cells compared to pcDNA (control) (i.e. pcDNA+pro-caspase-1+pro-IL-1β) transfected cells (Fig. 3A). There was an enhancement of IL-1β production by 110 pg/ml following expression of ASC in 293 cells (Fig. 3A). Expression of pro-IL-1β, caspase-1 and ASC in transfected 293 cells was confirmed by RT-PCR (data not shown). Pro-IL-1β and caspase-1 expression was similar in pro-IL-1β+pro-caspase-1+pcDNA transfected cells and pro-IL-1β+pro-caspase-1+ASC transfected 293 cells (data not shown). ). Similar to NLRP3, low levels of endogenous ASC was detected following RSV infection of 293 cells (data not shown) and ASC over-expression (following transfection with ASC plasmid) augmented IL-1β secretion from infected 293 cells.

Mouse primary BMDM that lacks ASC expression (derived from ASC KO mice) was utilized to further confirm the critical function of ASC during IL-1β secretion from RSV infected cells. IL-1β production was evaluated by ELISA analysis of medium supernatant derived from RSV infected WT and ASC KO cells. Similar to NLRP3, ASC is also a key player involved in IL-1β release. We observed significant loss of IL-1β secretion from infected ASC KO BMDM in comparison to infected WT BMDM (Fig. 3B). Based on these results and data shown in Fig. 2 we concluded that NLRP3/ASC inflammasome is indispensible for efficient IL-1β secretion during RSV infection.

### Both NLRP3 and ASC are required for caspase-1 activation following RSV infection

**Figure 4:**
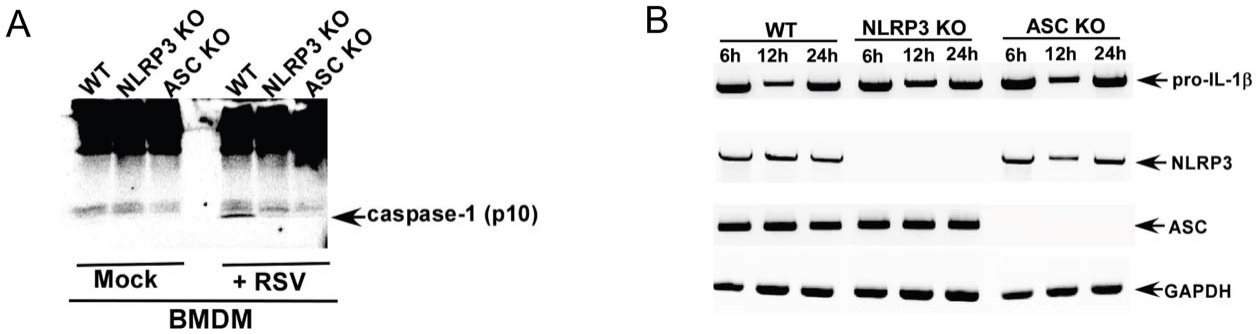
NLRP3 and ASC are required for caspase-1 activation following RSV infection. (**A**) Wild type (WT), NLRP3 knock-out (KO) and ASC KO BMDMs were infected with RSV (1 MOI) for 12 h. The cell lysate from mock infected and RSV infected cells were subjected to Western blot analysis with mouse caspase-1 p10 subunit specific antibody. (**B**) RT-PCR analysis of pro-IL-1β, ASC and NLRP3 expression in RSV infected WT, NLRP3 KO and ASC KO BMDMs. The gels shown in (A) and (B) is representative of three independent experiments that yielded similar results.

Inflammasome is required for caspase-1 activation and subsequent release of mature IL-1β. Since NLRP3/ASC inflammasome is required for IL-1β secretion (Fig. 2 and 3), we speculated that it is also required for caspase-1 activation during RSV infection. The hallmark of inflammasome complex mediated caspase-1 activation is cleavage of pro-casapase-1 into enzymatically active hetero-dimer of two p20 and p10 subunits. Therefore, detection of p10 subunit serves as an indicator of inflammasome complex mediated generation of active caspase-1. In order to investigate whether NLRP3/ASC is needed for caspase-1 activation, we infected WT, NLRP3 KO and ASC KO BMDMs with RSV. At 12 h post infection, the cell lysate was subjected to SDS-PAGE and Western blotting with anti-caspase-1 p10 subunit specific antibody. Western blot analysis revealed lack of p10 in infected NLRP3 KO and ASC KO BMDM compared to WT counterpart (Fig. 4A). Specificity of the p10 product is borne out by lack of any p10 subunit in mock infected WT cells and in KO cells. Equal loading was confirmed by re-probing the same blot after stripping with anti-actin antibody (Fig. S3). Thus, NLRP3/ASC inflammasome formation is an essential pre-requisite for caspase-1 activation in RSV infected cells. The important role of NLRP3/ASC inflammasome in caspase-1 activation was further confirmed by observing that reduction in IL-1β production from ASC KO and NLRP3 KO cells (Fig. 2B, 3B) were solely due to failure in caspase-1 activation, since RT-PCR analysis revealed similar expression of pro-IL-1β and inflammasome components (ASC or NLRP3) in RSV infected WT, NLRP3 KO and ASC KO cells. (Fig. 4B). Our studies have led to identification of ASC/NLRP3 inflammasome as the essential component required for caspase-1 activation and IL-1β secretion during RSV infection.

### NF-κB signaling is required for expression of pro-IL-1β and NLRP3 during RSV infection

**Figure 5:**
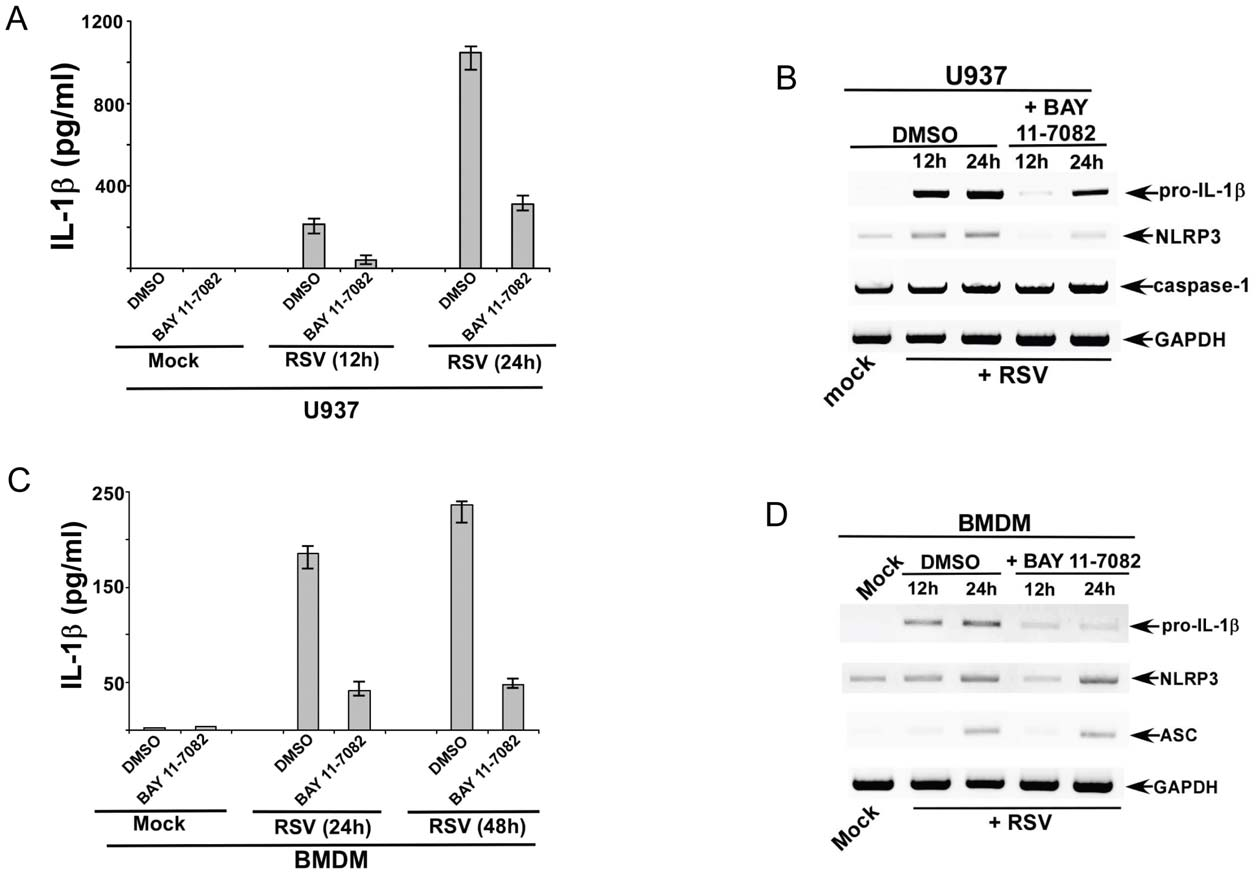
NF-κB signaling is critical for IL-1β secretion during RSV infection. (**A**) Human U937 cells were infected with RSV (1 MOI) in the presence of either DMSO (control) or NF-κB inhibitor (BAY 11-7082). IL-1β levels in the medium supernatant were assayed by ELISA at 12 h and 24 h post-infection. (**B**) RT-PCR analysis of pro-IL-1β, caspase-1 and NLRP3 expression in U937 cells infected with RSV in the presence of either DMSO (control) or BAY 11-7082. (**C**) Wild type primary mouse bone marrow derived macrophages (BMDM) were infected with RSV (1 MOI) in the presence of either DMSO (control) or BAY 11-7082. IL-1β levels in the medium supernatant were assayed by ELISA at 12 h and 24 h post-infection. (**D**) RT-PCR analysis of pro-IL-1β, ASC and NLRP3 expression in WT BMDMs infected with RSV in the presence of either DMSO (control) or BAY 11-7082. The gels shown in (B) and (D) are representative of three independent experiments that yielded similar results. For ELISA results, each value represents the mean ± standard deviation from three independent experiments.

The transcription factor NF-κB regulates diverse cellular processes including acting as a trans-activator of genes involved in innate immune response. NF-κB activation is required for IL-1β release following stimulus with various PAMPs and DAMPs [42]–[45]. It is envisioned that such function of NF-κB is manifested by various mechanisms including, expression of NLRP3 and pro-IL-1β genes [42]–[45]. RSV activates NF-κB during infection [40], [46]–[50]; however, the role of NF-κB in controlling IL-1β production during RSV infection has not been examined yet. In order to investigate whether NF-κB activation is required for IL-1β production, we inhibited NF-κB activation in infected cells by using a specific NF-κB inhibitor BAY 11-7082 [42]. U937 cells were pre-treated with DMSO (vehicle control) or BAY 11-7082 (5 µM) for 2 h, followed by RSV infection in the presence of either DMSO or BAY 11-7082. At 12 h and 24 h post-infection time-points, IL-1β levels in the medium supernatant were measured by ELISA assay. Indeed, NF-κB activation is required for IL-1β secretion during RSV infection, since significant loss of IL-1β production was observed upon blocking NF-κB activation (Fig. 5A). The NF-κB dependent mechanism of IL-1β production was further probed by investigating expression pro-IL-1β, caspase-1, and NLRP3 gene expression in BAY 11-7082 treated cells vs. control cells. For these studies, we treated U937 cells with either DMSO or BAY 11-7082 as discussed above. Total RNA isolated from RSV infected cells (+/− DMSO or BAY 11-7082) were used for RT-PCR analysis. Our results revealed that during RSV infection, activated NF-κB is required for expression of several key genes, including pro-IL-1β and NLRP3. Expression of pro-IL-1β and NLRP3 was drastically reduced in RSV infected NF-κB inhibited cells compared to control cells (Fig. 5B). In contrast, expression of caspase-1 was unaltered upon NF-κB inhibition.

The results with U937 cells were further verified using primary WT mouse BMDM. Similar to U937 cells, NF-κB activation was critical for IL-1β secretion from RSV infected BMDM. Treatment of BMDM with BAY 11-7082 resulted in diminished IL-1β production upon RSV infection (Fig. 5C). The loss of IL-1β release was due to reduced pro-IL-1β and NLRP3 expression in NF-κB inhibited cells. RT-PCR analysis revealed drastic reduction in pro-IL-1β and NLRP3 mRNA expression in RSV infected cells that were treated with BAY 11-7082 (Fig. 5D). Similar results (i.e. loss of IL-1β production and reduction in pro-IL-1β and NLRP3 gene expression) with U937 cells and BMDM were obtained by using another NF-κB specific inhibitor, IKK-2 inhibitor IV (Calbiochem) (data not shown). The endogenous basal mRNA levels of pro-IL-1β, NLRP3, ASC and caspase-1 was same in DMSO treated vs. BAY 11-7082 treated U937 cells and BMDMs (data not shown). The NF-κB inhibitors (at concentrations used in the above studies) were not toxic to the cells as cell viability assay did not show significant loss of cell viability during the time-period of the experiments described above (data not shown). Moreover, similar concentrations of BAY 11-7082 were utilized previously to assess function of NF-κB in macrophages [42]. Our studies have demonstrated that NF-κB is required for activation of pro-IL-1β and NLRP3 genes during RSV infection. Thus, NF-κB is a critical player during initial (signal-1, first signal) process of inflammasome activation and subsequent mature IL-1β secretion from RSV infected cells.

### TLR2/MyD88 pathway regulates pro-IL-1β and NLRP3 gene expression during RSV infection

During pathogen invasion the “first signal” compromises of activation of PRR(s) by PAMP(s) leading to NF-κB mediated expression of pro-IL-1β and inflammasome-associated (e.g. NLRP3) genes. TLRs are type-I integral membrane PRRs involved in induction of innate immune response against wide spectrum of pathogens. MyD88 serves as a major adaptor protein for TLR-mediated signal transduction. TLR stimulation by PAMPs culminates in activation of NF-κB, MAPK and IRF3 pathways resulting in expression of pro-inflammatory and antiviral genes. So far the function of TLRs as the “first signal” for IL-1β production has been only document for few pathogens including, Vibrio, Candida albicans, vaccinia virus, fungal components [51]–[55]. Since RSV activates TLR signaling (via TLR2 and TLR4) during infection [56]–[58], we next investigated whether TLR/MyD88 pathway is required for IL-1β secretion during RSV infection. Our result (Fig. 5) showing NF-κB mediated expression of pro-IL-1β and NLRP3 genes also formed the foundation for focusing our attention on the role of TLRs during IL-1β secretion from RSV infected cells.

**Figure 6:**
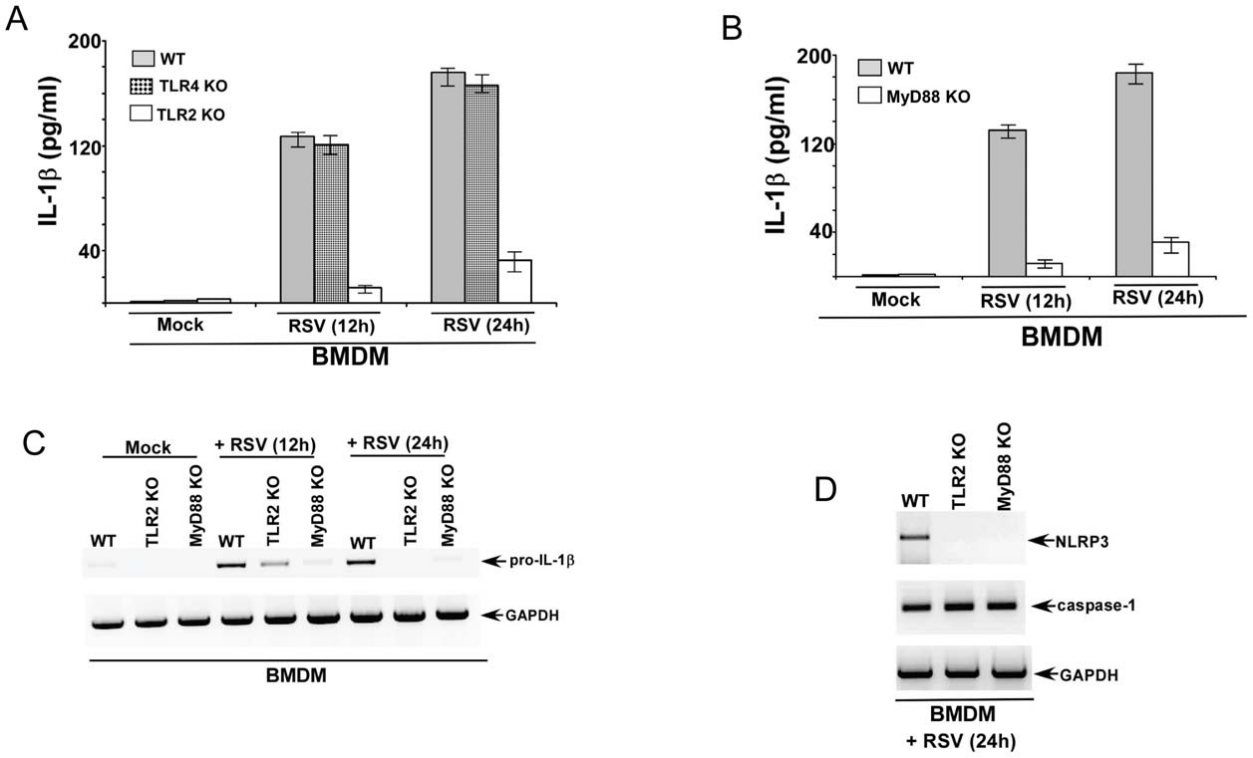
TLR2/MyD88 pathway is required for IL-1β release during RSV infection. (**A**) Wild type (WT), TLR2 knock-out (KO) and TLR4 KO BMDMs were infected with RSV (1 MOI). IL-1β levels in the medium supernatant were assayed by ELISA at 12 h and 24 h post-infection. (**B**) Wild type (WT) and MyD88 knock-out (KO) BMDMs were infected with RSV (1 MOI). IL-1β levels in the medium supernatant were assayed by ELISA at 12 h and 24 h post-infection. (**C**) RT-PCR analysis of pro-IL-1β expression in RSV infected WT, TLR2 KO and MyD88 KO BMDMs. (D) RT-PCR analysis of caspase-1 and NLRP3 expression in RSV infected WT, TLR2 KO and MyD88 KO BMDMs. The gels shown in (C) and (D) are representative of three independent experiments that yielded similar results. For ELISA assay, each value represents the mean ± standard deviation from three independent experiments.

In order to study the role of TLR pathway in IL-1β secretion, we utilized primary mouse BMDMs lacking TLR2, TLR4 and MyD88 expression. We decided to focus on TLR2/4 since these TLRs are activated by RSV in immune cells like macrophages [56]–[58] and previous studies have shown that TLR2/4 are involved in IL-1β secretion during infection with other pathogens [53]–[55]. BMDM prepared from WT, TLR2 KO, TLR4 KO and MyD88 KO mice were infected with RSV. At 12 h and 24 h post-infection time-points, medium supernatant was collected to assess IL-1β levels by ELISA. Surprisingly, our studies revealed that TLR2/MyD88 pathway is critical for IL-1β production; whereas the role of TLR4 during this process is minimal. We observed significantly reduced IL-1β levels in the medium supernatant derived from RSV infected TLR2 KO and MyD88 KO BMDM compared to infected WT and TLR4 BMDM (Fig. 6A, 6B).

The mechanism of TLR2/MyD88 pathway mediated IL-1β release was next examined. Based on our data showing NF-κB dependent pro-IL-1β and NLRP3 gene expression during RSV infection (Fig. 5); we postulated that activation of TLR2/MyD88 by RSV results in NF-κB activation and subsequent expression of essential genes like pro-IL-1β and NLRP3. Indeed, TLR2/MyD88 pathway is absolutely critical for pro-IL-1β and NLRP3 gene expression during RSV infection. RT-PCR analysis revealed drastic loss of pro-IL-1β (Fig. 6B) and NLRP3 (Fig. 6C) mRNAs in RSV infected TLR2 KO and MyD88 KO cells. In contrast, caspase-1 expression was unaltered in TLR2 KO and MyD88 KO BMDMs (Fig. 6C). Thus our studied have identified TLR2/MyD88/NF-κB pathway as the crucial “first signal” required for expression of pro-IL-1β and NLRP3 genes during RSV infection.

### ROS generated during RSV infection promotes IL-1β secretion

**Figure 7:**
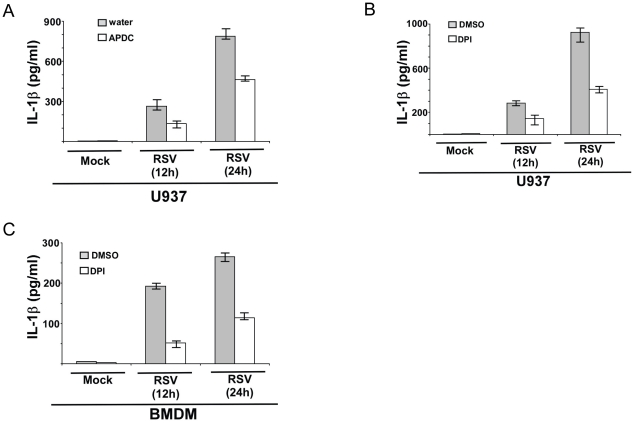
ROS produced during RSV infection triggers IL-1β secretion. (**A**) U937 cells were infected with RSV (1 MOI) in the presence of ROS inhibitor APDC (50 µM). Water served as the vehicle control. IL-1β levels in the medium supernatant were assayed by ELISA at 12 h and 24 h post-infection. (**B**) U937 cells were infected with RSV (1 MOI) in the presence of ROS inhibitor DPI (10 µM). DMSO served as the vehicle control. IL-1β levels in the medium supernatant were assayed by ELISA at 12 h and 24 h post-infection. (**C**) Wild type primary mouse bone marrow derived macrophages (BMDM) were infected with RSV (1 MOI) in the presence of ROS inhibitor DPI (2 µM) or DMSO (vehicle control). IL-1β levels in the medium supernatant were assayed by ELISA at 12 h and 24 h post-infection. Each value represents the mean ± standard deviation from three independent experiments.

Next, we investigated the “second signal” that initiates inflammasome assembly and release of mature IL-1β. First, we focused on the role of ROS in IL-1β production, since – a) ROS triggers NLRP3/ASC inflammasome formation during various conditions [14]–[19], [25], [29], [36], [59]–[65], b) ROS is required for NLRP3/ASC inflammasome mediated IL-1β secretion during influenza A virus infection [31], c) RSV infection results in intracellular ROS production [66], [67]. The role of ROS was examined by using ROS inhibitor DPI [68] and ROS scavenger APDC [25], [36]. Both these ROS inhibitors/scavengers were utilized previously to study NLRP3/ASC inflammasome dependent IL-1β production [25], [36], [68]. For our studies, we pre-treated U937 cells with DPI (10 µM) and APDC (50 µM) for 2 h, followed by RSV infection in the presence of the inhibitors or vehicle control (water for APDC and DMSO for DPI). At 12 h and 24 h post-infection, IL-1β levels in the medium supernatant were measured by ELISA. ROS play a vital role in IL-1β secretion during RSV infection, since treatment of cells with DPI and APDC lead to 41%–56% inhibition in IL-1β release from RSV infected cells (Fig. 7A, 7B).

The role of ROS was further verified using primary BMDM. Initially we incubated BMDM with 10 µM DPI during RSV infection. However, we observed significant decrease in NLRP3 and IL-1β expression following 10 µM DPI treatment (data not shown). This result was similar to that was published recently [68], whereby 10 µM-20 µM DPI significantly reduced expression of pro-IL-1β and NLRP3 genes in BMDM. In contrast, it was shown that 1 µM-2 µM DPI did not effect NLRP3 expression and IL-1β protein levels in BMDM [68]. Therefore, we treated BMDM with 2 µM DPI, which did not alter NLRP3 gene expression and intracellular IL-1β levels (data not shown). Incubation of WT BMDM with DPI (2 µM) resulted in significant reduction (by 56%–70%) in IL-1β production upon RSV infection, compared to control (DMSO treated) cells (Fig. 7C). Please note that DPI (2 µM) and APDC (50 µM) concentrations used in the current study is similar (or lower) compared to concentrations used to in previous studies dealing with the role of ROS in inflammasome activation in macrophages [25], [68]. We have thus identified ROS as one of the crucial intracellular signal that promotes inflammasome activation and IL-1β release from RSV infected cells.

### IL-1β release during RSV infection requires potassium efflux by ATP-sensitive potassium channel

**Figure 8:**
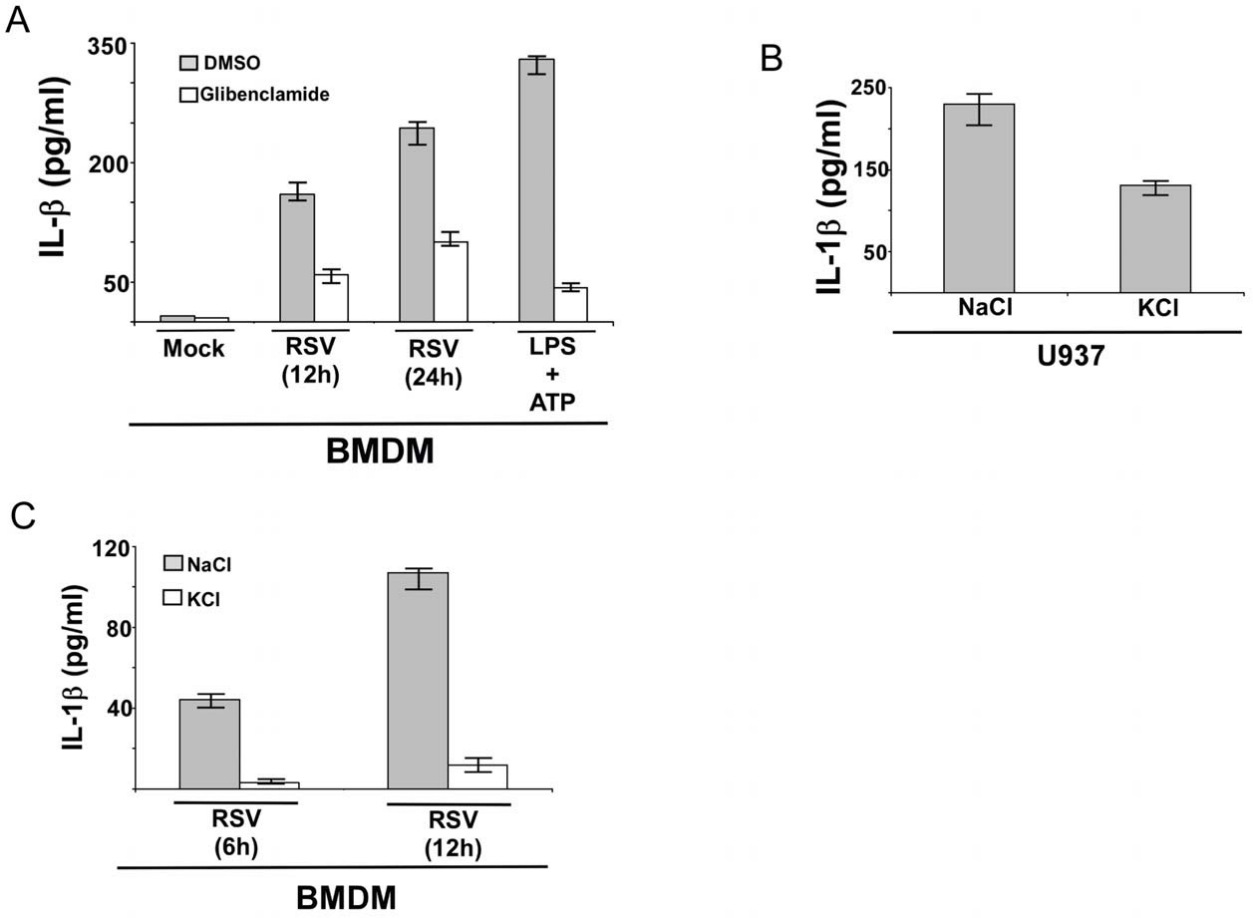
Potassium efflux plays a critical role in IL-1β production during RSV infection. (**A**) Wild type primary mouse bone marrow derived macrophages (BMDM) were infected with RSV (1 MOI) in the presence of ATP-sensitive potassium channel inhibitor glibenclamide (50 µM). DMSO served as the vehicle control. IL-1β levels in the medium supernatant were assayed by ELISA at 12 h and 24 h post-infection. Cells were also treated with LPS and ATP in the presence of either DMSO or glibenclamide. (**B**) U937 cells were infected with RSV (1 MOI) in the presence of buffer containing either NaCl (150 mM) (control) or KCl (150 mM). IL-1β levels in the medium supernatant were assayed by ELISA at 12 h post-infection. (**C**) BMDM were infected with RSV (1 MOI) in the presence of buffer containing either NaCl (150 mM) (control) or KCl (150 mM). IL-1β levels in the medium supernatant were assayed by ELISA at 6 h and 12 h post-infection. Each value represents the mean ± standard deviation from three independent experiments.

Since loss of ROS did not completely abrogate IL-1β production following RSV infection (Fig. 7), we speculated that additional mechanism is required for inflammasome activation. Since RSV infection results in extracellular release of ATP and UTP [69], [70], we postulated that potassium efflux by ATP-sensitive potassium channel may constitute an additional “second signal”. In order to study the contribution of potassium efflux in IL-1β production, we inhibited ATP-sensitive potassium channel with glibenclamide. We used non-toxic concentration (50 µM) of glibenclamide as previously reported for studying the role of ATP-sensitive potassium channel in inflammasome activation in macrophages [25], [71], [72]. WT BMDMs were pretreated with glibenclamide (50 µM) for 1 h, followed by RSV infection in the presence of glibenclamide or DMSO (vehicle control). IL-1β levels in the medium supernatant were measured by ELISA at 12 h post-infection. As expected, glibenclamide dramatically blocked (by 88%) IL-1β release from LPS/ATP treated cells (Fig. 8A). ATP-sensitive potassium channel is also required for IL-1β secretion during RSV infection, since significantly less (inhibited by approximately 60%) IL-1β was released from infected cells treated with glibenclamide (Fig. 8A). Glibenclamide treatment did not alter expression of pro-IL-1β, NLRP3 and ASC gene expression, indeed pro-IL-1β and NLRP3 expression was enhanced in glibenclamide treated cells (Fig. S4A).

To further validate a role of potassium efflux in inflammasome activation, we next infected U937 cells (Fig. 8B) or WT BMDM (Fig. 8C) with RSV for 6 h and 12 h in the presence of buffer containing either 150 mM NaCl (control) or 150 mM KCl [61]. This method was previously utilized for blocking potassium efflux to study its role during silica mediated inflammasome activation [61]. These experiments were performed for shorter infection time period (6 h–12 h post-infection vs. 12 h–24 h post-infection) since prolonged exposure of cells to NaCl and KCl may induce cellular toxicity. As shown in Fig. 8B, incubation with excess potassium (i.e. with medium containing 150 mM KCl) resulted in significant reduction (by 44%) in IL-1β release from RSV infected U937 cells. Drastic loss of IL-1β release (inhibition of IL-1β secretion by 90%) was also noted in RSV infected BMDM incubated with 150 mM KCl buffer (Fig. 8C). As expected dramatic loss (inhibition by 95%) of IL-1β was observed from LPS and ATP treated BMDM incubated with KCl buffer (Fig. S4B). These studies have demonstrated that both ROS and potassium efflux (mediated by ATP-sensitive potassium channel) is required for inflammasome assembly and caspase-1 activation, leading to mature IL-1β secretion from RSV infected cells.

## Discussion

Innate immunity comprises the first line of defense against invading pathogens [4]–[7]. IL-1β is a pyrogenic cytokine that is produced during innate immune response following pathogenic invasion. IL-1β plays a very important role in shaping the inflammatory response against pathogens. Restricted inflammation is required to clear the pathogen and launch an effective adaptive immune response. IL-1β is synthesized as an immature pro-form (pro-IL-1β) which requires activated caspase-1 for cleavage into mature IL-1β that is secreted from the cells. Extracellular IL-1β confers its pro-inflammatory effect via an autocrine/paracrine mechanism following binding to its cognate receptor, IL-1βR.

Multimeric protein complex called inflammasome is indispensible for caspase-1 activation (14–37). One of the major constituent of the inflammasome complex is the NLR family proteins. Recently, AIM2 was identified as an important non-NLR inflammasome required for caspase-1 activation [73]. There is also a report of RIGI/CARD-9 mediated inflammasome activation [74]. The assembly of specific type of inflammasome is dictated by the nature of stimuli (or signal). Recent studies have validated the emergence of inflammasome as a vital component regulating health and disease [14]–[37]. Genetic disorders involving activation of NLRP3 inflammasome is associated with various inflammatory disorders. Host derived damage-associated molecular patterns (DAMPs) and endogenous molecules also activate inflammasome, which culminates in various disease states. For example, ATP, monosodium urate (MSU), cholesterol crystals, amyloid beta, hyaluronan, islet amyloid polypeptide (IAPP), fatty acid and glucose activates NLRP3 inflammasome [14]–[37], which contributes to disease associated with these DAMPs/molecules – i.e. MSU (gout), amyloid beta (Alzheimer's disease), cholesterol crystals (atherosclerosis), IAPP and glucose (diabetes), fatty acid (type II diabetes), hyaluronin (inflammation during tissue damage) and ATP (chronic and atopic inflammation in the respiratory tract). Apart from these diseases, exposure to environmental pollutants like asbestos and silica activates NLRP3 inflammasome to cause exaggerated inflammatory response in the airway. These extensive studies have illustrated an essential function of inflammasome components (e.g. NLRP3) in regulating patho-physiological condition associated with cellular and tissue homeostasis.

Infection with various pathogens (virus, bacteria, fungus, parasites) also triggers inflammasome formation. Controlled pro-inflammatory response is needed for pathogen clearance and induction of an effective adaptive immunity. Both DNA and RNA viruses utilizes various inflammasomes for caspase-1 activation and IL-1β secretion. DNA viruses like vaccinia virus and mouse cytomegalovirus utilizes AIM2/ASC inflammasome for caspase-1 activation and IL-1β release [75]. NLRP3 inflammasome serve as the platform for caspase-1 activation (and IL-1β release) during infection with both DNA (adenovirus, herpes virus, vaccinia virus) and RNA (influenza A virus, mouse Sendai virus, mouse encephalomyocarditis virus) viruses [34]. Among the human lung-tropic viruses, a critical role of NLRP3 [30], [31] and ASC [32] inflammasome in controlling influenza A virus infection and pathogenesis was illuminated by several studies. NLRP3/ASC inflammasome confers optimal airway inflammation required for influenza A virus clearance from the respiratory tract.

In infected macrophages, two discrete signals are required for inflammasome formation and caspase-1 activation [38] – a) First signal (signal-1) constitutes gene expression of pro-IL-1β and components of inflammasome complex (e.g. NLRP3); which is mediated by induction of cellular signaling pathways (e.g. NF-κB pathway) following PRR (e.g. TLRs) activation by PAMPs (e.g. LPS), and b) second signal (signal-2) triggers assembly of inflammasome complex. The second signal is relayed by various mechanism including, generation of intracellular ROS, potassium efflux due to stimulation of ATP-sensitive potassium channel or pore formation by bacterial toxins, lysosomal disruption leading to cathepsin B leakage into the cytoplasm [14]–[19], [29]. Interestingly, ROS may also confer first signal by regulating pro-IL-1β and NLRP3 gene expression [68]. Among the human respiratory viruses, mechanism of inflammasome formation is only characterized following influenza A virus infection [30]–[32], [76]. Although, the nature of first signal (required for pro-IL-1β and NLRP3 gene expression) during influenza A virus infection is still unknown, there is evidence for the existence of “first signal” during influenza A virus infection, since *in vivo* (in mice) and *in vitro* infection with influenza A virus resulted in induction of both pro-IL-1β and NLRP3 gene expression [31]. At least two second signals including ROS and endosomal ion flux (mediated by influenza A virus M2 ion channel protein) has been implicated in triggering inflammasome assembly flowing influenza A virus infection [31], [76]. In that context, our current studies have elucidated both the first and second signals required for inflammasome mediated IL-1β release during infection with another clinically important human respiratory virus, RSV.

**Figure 9:**
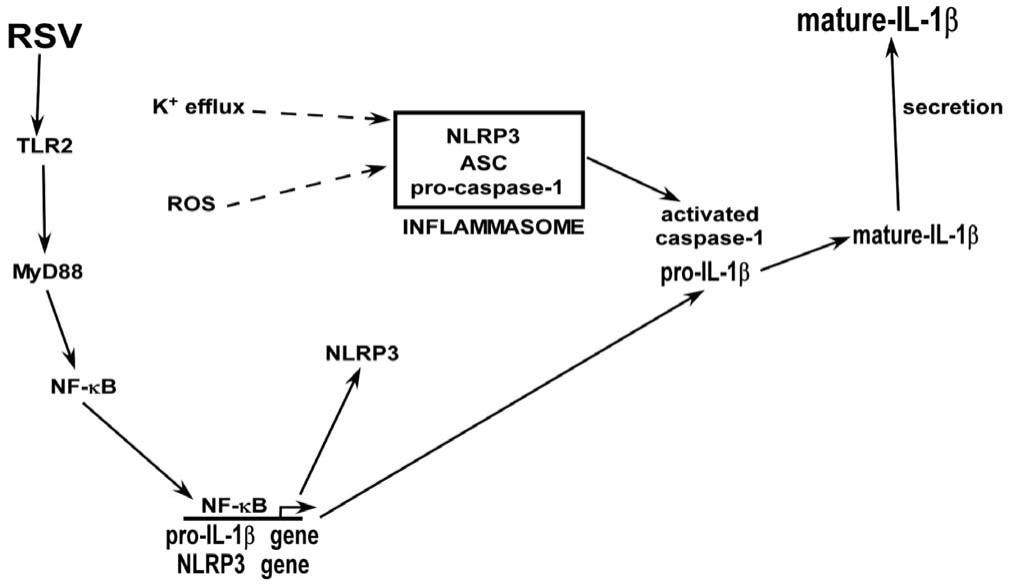
A schematic model depicting the mechanism of IL-1β secretion during RSV infection. RSV infection activates TLR2/MyD88 pathway that culminates in NF-κB activation. Nuclear translocation of NF-κB results in NF-κB mediated trans-activation of pro-IL-1β and NLRP3 genes. Intracellular ROS generated during RSV infection and potassium efflux due to stimulation of ATP-sensitive ion channel triggers assembly of NLRP3/ASC inflammasome complex. NLRP3/ASC inflammasome activates caspase-1, which subsequently cleaves pro-IL-1β protein into its mature form. Mature IL-1β is secreted from the cells.

Similar to influenza A virus (a orthomyxovirus), RSV (a paramyxovirus) is a single-stranded RNA respiratory virus that causes mortality and morbidity among children, elderly and immuno-compromised individuals [1]–[3]. Although RSV infection results in IL-1β secretion [9]–[12], so far the mechanism is not known. Here in, we have characterized the mechanism involved in IL-1β secretion during RSV infection. Our results demonstrated that – a) activated caspase-1 is required for IL-1β secretion, b) NLRP3/ASC inflammasome complex activates caspase-1 during RSV infection, c) TLR2/MyD88 pathway mediated NF-κB activation is essential for expression of pro-IL-1β and NLRP3 genes (first signal), and d) ROS and potassium efflux (via stimulation of ATP-sensitive potassium channel) generated in RSV infected cells serves as the second signal for inflammasome complex assembly. Based on these results we propose a model (Fig. 9) whereby RSV infection induces TLR2/MyD88 pathway, leading to activation of NF-κB. NF-κB translocates to the nucleus to trans-activate pro-IL-1β and NLRP3 genes. ROS and potassium efflux (via stimulation of ATP-sensitive potassium channel) generated in infected cells trigger formation of NLRP3/ASC inflammasome complex; which cleaves pro-caspase-1 to generate activate caspase-1. Cleavage of pro-IL-1β protein by activated caspase-1 results in secretion of mature functional IL-1β. It is important to mention that as recently noted [68], ROS may also serve as a “first signal” during RSV infection, since high concentration of ROS inhibitor reduced expression of pro-IL-1β and NLRP3 gene expression during RSV infection (data not shown). The mechanism by which ROS is regulating gene expression needs further characterization. Nevertheless, using ROS inhibitor concentration that did not affect NLRP3 and pro-IL-1β levels, we demonstrated that ROS could also function as a “second signal” following RSV infection. Our results have illustrated that similar to influenza A virus [30]–[32], RSV also utilizes NLRP3/ASC inflammasome for caspase-1 activation and ROS is required during this event. However, we have extended our studies to document that TLR2/MyD88/NF-κB pathway is essential for expression of pro-IL-1β and NLRP3 genes (first signal). In addition, two second signals (ROS and potassium efflux) are required to promote inflammasome formation following RSV infection. Thus, RSV is capable of utilizing two different PRRs (membrane bound PRRs like TLR2 and cytoplasmic PRR like NLRP3) to orchestrate mature IL-1β release via a NF-κB dependent process.

The pre-requisite for IL-1β release constitute optimal expression of pro-IL-1β and inflammasome related (e.g. NLRP3) genes (first signal), which precedes inflammasome complex formation. So far the first signal has not been widely characterized. Few studies have suggested that PAMP or DAMP mediated activation of membrane bound TLRs transduce signal to activate NF-κB for expression of pro-IL-1β and inflammasome-related gene like NLRP3 [42]–[45], [51]–[55]. TLRs/MyD88 was required for inflammasome assembly after infection [vaccinia virus (TLR2/6), Candida albicans (TLR2), Vibrio (MyD88)] and stimulation with DAMPs [biglycan (TLR2/4)], environmental particles [alkane particles (TLR2)] or fungal components [zymosan and mannan (TLR2)] [51]–[55], [77]. Activated NF-κB was necessary for inflammasome formation during infection (Streptococcus pyogenes, Vibrio) [45], [51] or stimulation with various ligands/agents (TLR4 ligand LPS, TLR2/1 ligand Pam3CysSK4, silica) [42], [43]. Specifically, TLR/NF-κB pathway was essential for expression of pro-IL-1β and NLRP3 genes during various conditions [42]–[44], [51]. Although RNA viruses (e.g. influenza A virus) utilize inflammasome for caspase-1 activation, the “first signal” associated with IL-1β release has not been elucidated. In that regard, we report that TLR2/MyD88/NF-κB pathway serves as a crucial first signal for pro-IL-1β and NLRP3 gene expression during RSV infection.

RSV utilizes both TLR4 and TLR2 for launching a pro-inflammatory response during RSV infection of the macrophages [56]–[58]. In corroboration with previous studies, we identified TLR2/MyD88/NF-κB pathway as the major signaling component responsible for expression of pro-IL-1β and NLRP3 genes following RSV infection of macrophages. The specificity of this pathway is borne out by the observation that TLR4 was not involved during this process. This observation was surprising, since TLR4 was also identified as one of the PRRs involved in pro-inflammatory response during RSV infection of macrophages [56], [57]. However, TLR2 dependent response was predominant in RSV infected macrophages compared to TLR4 [58]. Although TLR2 may serve as the major PRR, other PRRs or mechanisms could contribute to NF-κB mediated expression of pro-IL-1β and NLRP3 genes, since RSV infection of TLR2 KO and MyD88 KO cells did not completely abrogate IL-1β production. Moreover, apart from NF-κB, MAPK pathways may also play a role in expressing pro-IL-1β and NLRP3 genes because pro-IL-1β and NLRP3 gene possess AP-1 binding site in its promoter region (unpublished observation) and both RSV and TLR2 induce MAPK pathway [78], [79]. In the future we will investigate the precise mechanism by which TLR2/MyD88 pathway regulate pro-IL-1β and NLRP3 gene expression during RSV infection.

Various mediators transmit the second signal for inflammasome complex assembly. This signal is conferred by intracellular ROS, potassium efflux, cytoplasmic cathepsin B (leakage of cathepsin B due to lysosomal disruption). During influenza A virus infection, ROS and endosomal ion flux (mediated by viral M2 proton channel) constitute the second signal for inflammasome formation [31], [76].

Our studies have illustrated that ROS produced during RSV infection and potassium efflux due to stimulation of ATP-sensitive potassium channel serve as the second signal to promote inflammasome activation. RSV infection results in generation of oxygen radicals like ROS [66], [67]. Scavenging or inhibiting intracellular ROS substantially reduced IL-1β secretion. ROS has been implicated in caspase-1 activation during various patho-physiological conditions like diabetes; ROS production was essential for inflammasome formation by glucose, IPP and fatty acids [25], [36], [63]. Intracellular ROS is also critical for inflammasome activity following exposure to pollutants (asbestos and silica) [60], [61] and infection with viruses (influenza A virus, adenovirus) [31], [65], fungus (Aspergillus fumigatus) [64] and parasites (malarial hemozoin) [59]. It is also reported that mitochondrial ROS (in contrast to ROS generated by NADPH oxidases or NOX) is involved in inflammasome activation [80], [81]. In that context, we have used two ROS inhibitor/scavenger (APDC and DPI) that is known to block NOX derived ROS and ROS originating from mitochondria. Recently, it was shown that high concentrations (10 µM–20 µM) of ROS inhibitors (DPI and NAC) significantly diminished expression of pro-IL-1β and NLRP3 genes in mouse macrophages [68]. Indeed, we also observed loss of pro-IL-1β and NLRP3 mRNA expression by using high concentrations (i.e. 10 µM) of ROS inhibitor DPI during RSV infection (data not shown). However, to assess the role of ROS during second signal, we have used low (2 µM) concentrations of DPI. As previously noted [68], 1 µM–2 µM DPI did not affected NLRP3 gene expression and IL-1β protein levels in murine macrophages [68].

Since ROS scavengers did not completely abrogate IL-1β secretion, we speculated that additional mechanism(s) may contribute to inflammasome formation and caspase-1 activation during RSV infection. Efflux of potassium is known to activate inflammasome. Potassium efflux is achieved either by activation of ATP-gated ion channel P2X7R by extracellular ATP or pore formation by bacterial toxins [71], [72], [82]–[91]. Toward that end we have identified potassium efflux due to stimulation of ATP-sensitive ion channel as the additional second signal for IL-1β release during RSV infection. Potassium efflux contributes to inflammasome activation during various patho-physiological conditions like infection with bacteria (Staphylococcus aureus, Escherichia coli, Chlamydia, bacterial toxins) 71,82–87 or fungus [Candida albacans, β-glucan (major cell wall components of fungi)] [88], [89], diabetes [25], [36], exposure to silica [90] and treatment with antibiotics [91]. Role of intracellular ionic environment alteration (i.e. potassium efflux) in inflammasome activation during virus infection has not been reported. In that context, our study has illuminated potassium efflux as the second signal required by RSV for inflammasome activation. Although cathepsin B leakage due to lysosomal disintegration also activates inflammasome [14]–[19], [29], that phenomenon may not be occurring during RSV infection. Cathepsin B mediated inflammasome activation is only noted for crystalline and particulate molecules that activate inflammasome following cellular uptake leading to lysosomal destabilization [14]–[19], [29]. Moreover, lysosomal integrity is maintained during RSV infection (data not shown). Thus our studies have identified both ROS and potassium efflux as the key second signals essential for inflammasome activation during RSV infection. It is interesting to note that similar to RSV, there is precedence for inflammasome activation by two second signals. For example, β-glucan, candida albican and silica mediated inflammasome activation requires both ROS and potassium efflux [88]–[90].

Please note that we failed to detect significant difference in RSV infectivity (examined by performing plaque assay to determine viral titer) in control cells vs. cells treated with various inhibitors (i.e. ROS inhibitors and ATP-sensitive potassium channel inhibitor) (data not shown). Similarly, no difference in RSV titer was observed in WT vs. KO cells (i.e. NLRP3 KO, caspase-1 KO and ASC KO BMDMs) (data not shown).

In summary, we have characterized the mechanism of IL-1β secretion during RSV infection. IL-1β release during infection requires NLRP3/ASC inflammasome complex that activates caspase-1, the enzyme required for processing pro-IL-1β into mature IL-1β. TLR2/MyD88/NF-κB pathway serves as the first signal for expression of pro-IL-1β and NLRP3 genes. The second signal comprising of both ROS and potassium efflux promotes inflammasome activation.

## Materials and Methods

### Virus and cells

RSV (A2 strain) was propagated in CV-1 cells [40], [46]–[48]. RSV was purified by centrifugation (two times) on discontinuous sucrose gradients as described previously [92]. 293 cells were maintained in DMEM supplemented with 10% fetal bovine serum (FBS), penicillin, streptomycin, and glutamine. U937 cells were maintained in RPMI 1640 medium supplemented with 10% FBS, 100 IU/mL Penicillin, 100 µg/mL Streptomycin, 1 mM sodium pyruvate and 100 nM HEPES. Bone marrow-derived macrophages (BMDMs) were obtained from femurs and tibias of wild-type WT, ASC knock-out (KO), NLRP3 KO, TLR2 KO, TLR4 KO and MyD88 KO mice and were cultured for 6–8 days as described earlier [40], [93]. Cells were plated on 6-well plates containing RPMI, 10% FBS, 100 IU/mL Penicillin, 100 µg/mL Streptomycin and 20 ng/ml GM-CSF. Femurs and tibia (for isolation of BMDM) were provided by our collaborator Dr. Michael Berton (University of Texas Health Science Center), who procured the TLR2 KO, TLR4 KO and MyD88 KO mice from Dr. Doug Golenbock (University of Massachusetts Medical School, Worcester, MA) under a Materials Transfer Agreement with Dr. Shizuo Akira (Osaka University, Osaka, Japan). 293 and U937 cells were obtained from American Type Culture Collection (ATCC), Manassas, VA.

### RNA isolation, PCR amplification and reverse transcription-PCR (RT-PCR)

Total RNA isolation from cells was performed using a monophasic solution of phenol and guanidine thiocyanate (TRIzol) (Sigma-Aldrich, St. Louis, MO), as recommended by the suppliers. Total cellular RNA (∼1 µg) was used to generate cDNA using Moloney murine leukemia virus reverse transcriptase (Applied Biosystems, Carlsbad, CA). PCR was routinely performed using 0.25 units of *Taq* polymerase, 10 pmol of each oligonucleotide primer, 1 mM MgCl_2_, and 100 µM deoxynucleotide triphosphates in a final reaction volume of 25 µl. The amplification cycle was as follows: An initial denaturing step (95°C for 3–5 min) was followed by either 25, 30 or 35 cycles of denaturing (94°C for 30 sec), annealing (60°C for 30 s), and extending (72°C for 30 sec), followed by either 5 or 10 min at 72°C for elongation. Following amplification, the PCR products were analyzed on 2% agarose gels and band intensities were quantified by densitometry (Syngene gel-documentation system). Equal loading in each well was confirmed by analyzing expression of the housekeeping gene glyceraldehyde-3-phosphate dehydrogenase (GAPDH). The primers used to detect the indicated genes by RT-PCR are shown below.

Human ASC- Forward (5′-3′) GGACGCCTTGGCCCTCACCG, Reverse (5′-3′) GGCGCGGCTCCAGAGCCCTG (product size −150 bp)

Human NLRP3- Forward (5′-3′) TTCTTTCTGTTTGCTGAGTTTTTG, Reverse (5′-3′) TTCCTGGCATATCACAGTGG (product size −467 bp)

Human caspase-1- Forward (5′-3′) ATCCGTTCCATGGGTGAAGGTACA, Reverse (5′-3′) CAAATGCCTCCAGCTCTGTA (product size −616 bp)

Human pro-IL-1β- Forward (5′-3′) AAACAGATGAAGTGCTCCTTCCAG, Reverse (5′-3′) TGGAGAACACCACTTGTTGCTCCA (product size −391 bp)

Human GAPDH- Forward (5′-3′) GTCAGTGGTGGACCTGACCT, Reverse (5′-3′) AGGGGTCTACATGGCAACTG (product size −420 bp)

Mouse ASC- Forward (5′-3′) GTGGACGGAGTGCTGGATG, Reverse (5′-3′) GTCCATCACCAAGTAGGGATG (product size −189 bp)

Mouse NLRP3- Forward (5′-3′) CTGAAGATGACGAGTGTCCGTT, Reverse (5′-3′) ATGCTGCAGTTTCTCCAAGGCT (product size −1074 bp)

Mouse Caspase-1- Forward (5′-3′) TCCAGGAGGGAATATGTGG, Reverse (5′-3′) CTTGTTTCTCTCCACGGCA (product size −156 bp)

Mouse pro-IL-1β- Forward (5′-3′) GACAGTGATGAGAATGACCTGTTC, Reverse (5′-3′) CCTGACCACTGTTGTTTCCC (product size −730 bp)

Mouse GAPDH- Forward (5′-3′) GCCAAGGTCATCCATGACAACTTTGG, Reverse (5′-3′) GCCTGCTTCACCACCTTCTTGATGTC (product size −314 bp)

### ELISA assay for IL-1β

Medium supernatant collected from RSV infected macrophages or 293 cells were analyzed for IL-1β levels by using human or mouse IL-1β specific ELISA kit (eBioscience, San Diego, CA).

### 293 cell transfection

293 cells were transfected (Lipofectamine 2000 from Invitrogen was used for transfection) at 80% confluence [40] with expression plasmids encoding human pro-IL-1β (0.1 µg/well of 12-well plate), pro-caspase-1 (1.5 µg/well of 12-well plate), ASC (1 µg/well of 12-well plate), NLRP3 (1 µg/well of 12-well plate) or pcDNA6.1 (1 µg/well of 12-well plate). At 24 h post-transfection, 293 cells were infected with RSV (1 MOI).

### Viral infection of cells

293 cells, U937 cells and BMDM were infected with purified RSV at 1 multiplicity of infection (MOI) in serum free antibiotic free OPTI-MEM medium (GIBCO). Virus adsorption was performed for 1.5 h at 37°C. For 293 cells, following adsorption, cells were washed twice with serum containing DMEM and the infection was continued in the presence of serum containing DMEM for the specified time points. For U937 cells, following adsorption, cells were washed twice with serum containing RPMI medium and infection was continued in RPMI medium supplemented with 10% FBS, 100 IU/mL Penicillin, 100 µg/mL Streptomycin, 1 mM sodium pyruvate and 100 nM HEPES. For BMDM, following adsorption, cells were washed twice with serum containing RPMI medium and infection was continued in RPMI medium containing 10% FBS, 100 IU/mL Penicillin, 100 µg/mL Streptomycin and 20 ng/ml GM-CSF.

In some experiments, U937 cells and WT BMDM were pre-treated (for 2 h) with either caspase-1 inhibitors [Ac-(NMe)Tyr-Val-Ala-Asp-CHO (Ac-YVAD-CHO) (10 µM) (AnaSpec, Fremont, CA)], NF-κB inhibitor BAY 11-7082 (5 µM) (Sigma-Aldrich, St. Louis, MO), ROS inhibitors/scavengers [(2R,4R)-4 Aminopyrrolidine-2,4-dicarboxylic acid (APDC) (50 µM) (Enzo Life sciences, Plymouth Meeting, PA) or Diphenyleneiodonium Chloride (DPI) (10 µM or 2 µM) (EMD Bioscienes, Rockland, MA)], ATP-sensitive potassium channel inhibitor Glibenclamide (50 µM) (Sigma-Aldrich, St. Louis, MO). After pre-treatment, cells were infected with RSV in the absence or presence of the corresponding inhibitors. As a vehicle control either DMSO or water was used. For the experiment with glibenclamide cells were treated with LPS+ATP as a positive control. Cells were primed with LPS (5 µg/ml) (Invivogen, San Diego, CA) followed by addition of ATP (5 mM) (Sigma-Aldrich, St. Louis, MO) in the presence of DMSO or glibenclamide.

For some experiments, cells were infected in the presence of 150 mM KCl or 150 mM NaCl. U937 cells or BMDMs were pre-treated for 30 min with a buffer containing either 150 mM KCl (10 mM HEPES, 5 mM NaH_2_PO_4_, 150 mM KCl, 1 mM MgCl_2_, 1 mM CaCl_2_, 1% BSA, pH 7.4) or 150 mM NaCl (10 mM HEPES, 5 mM KH_2_PO_4_, 150 mM NaCl, 1 mM MgCl_2_, 1 mM CaCl_2_, 1% BSA, pH 7.4). Following pretreatment, cells were infected with purified RSV at 1 MOI in serum-free antibiotic free OPTI-MEM I. Following adsorption for 1 h at 37°C, OPTI-MEM containing RSV was replaced with fresh buffer containing either 150 mM KCl or 150 mM NaCl. At 6 h and 12 h post-infection, the medium supernatant was collected to measure IL-1β protein levels by ELISA. Cells were treated with LPS+ATP as a positive control. BMDM incubated with buffer containing either 150 mM NaCl or 150 mM KCl were primed with LPS (5 µg/ml) (Invivogen, San Diego, CA) for 12 h followed by addition of ATP (5 mM) (Sigma-Aldrich, St. Louis, MO). Following 30 min ATP treatment, medium supernatant was collected to measure IL-1β protein levels by ELISA.

### Caspase-1 p10 subunit Western blotting

Total cell lysate collected from RSV infected cells were used for Western blotting. Cell lysate (50 µg protein) was subjected to 12% SDS-PAGE. The separated proteins were transferred to PVDF membrane and blotted with antibody specific for either human or mouse caspase-1 p10 subunit. Both the human and mouse p10 antibodies were obtained from Santa Cruz Biotechnology (Santa Cruz, CA). In some experiment the blot was stripped with Western blot stripping buffer (Thermo Scientific, Rockford, IL) and re-probed with anti-actin antibody (Bethyl Laboratories, Montgomery, TX).

## Supporting Information

Figure S1 **(A) RT-PCR analysis of caspase-1 expression in mock and RSV infected 293 cells.** Caspase-1 expression was also monitored in RSV infected (at 24 h post-infection) 293 cells transfected with pro-caspase-1 plasmid (pro-caspase-1 transfected). **(B)** RT-PCR analysis of pro-IL-1β expression in mock and RSV infected 293 cells. Pro-IL-1β expression was also monitored in RSV infected (at 24 h post-infection) 293 cells transfected with pro-IL-1β plasmid (pro-IL-1β transfected). **(C)** RT-PCR analysis of NLRP3 expression in mock and RSV infected 293 cells. NLRP3 expression was also monitored in RSV infected (at 24 h post-infection) 293 cells transfected with NLRP3 plasmid (NLRP3 transfected).
(TIF)

Figure S2 **Primary normal human bronchial epithelial (NHBE) cells were infected with RSV (1 MOI) in the presence of either water (vehicle control) or caspase-1 inhibitor (10 µM of Ac-YVAD-CHO).** IL-1β levels in the medium supernatant were assayed by ELISA at 12 h post-infection. Each value represents the mean ± standard deviation from three independent experiments.
(TIF)

Figure S3 **Wild type (WT), NLRP3 knock-out (KO) and ASC KO BMDMs were infected with RSV (1 MOI) for 12 h.** The cell lysate from mock infected and RSV infected cells were subjected to Western blot analysis with mouse caspase-1 p10 subunit specific antibody (as shown in Fig. 4A). The blot was stripped and re-probed with anti-actin antibody.
(TIF)

Figure S4 **(A) RT-PCR analysis of pro-IL-1β, ASC, and NLRP3 expression in RSV infected wild type primary mouse bone marrow derived macrophages (BMDM) treated with either DMSO or glibenclamide (Gliben) (50 µM).** **(B)** WT BMDMs were treated with LPS+ATP (primed with LPS for 12 h, followed by stimulation with ATP for 30 mins) in the presence of buffer containing either 150 mM NaCl (control) or 150 mM KCl. IL-1β levels in the medium supernatant were assayed by ELISA. Each value represents the mean ± standard deviation from three independent experiments.
(TIF)
