# Supplementary figures and images for: TLR2/MyD88/NF-κB Pathway, Reactive Oxygen Species, Potassium Efflux Activates NLRP3/ASC Inflammasome during Respiratory Syncytial Virus Infection
Source: PLoS One. 2012 Jan 25;7(1):e29695. doi: 10.1371/journal.pone.0029695 (PMC3266238; doi:10.1371/journal.pone.0029695)

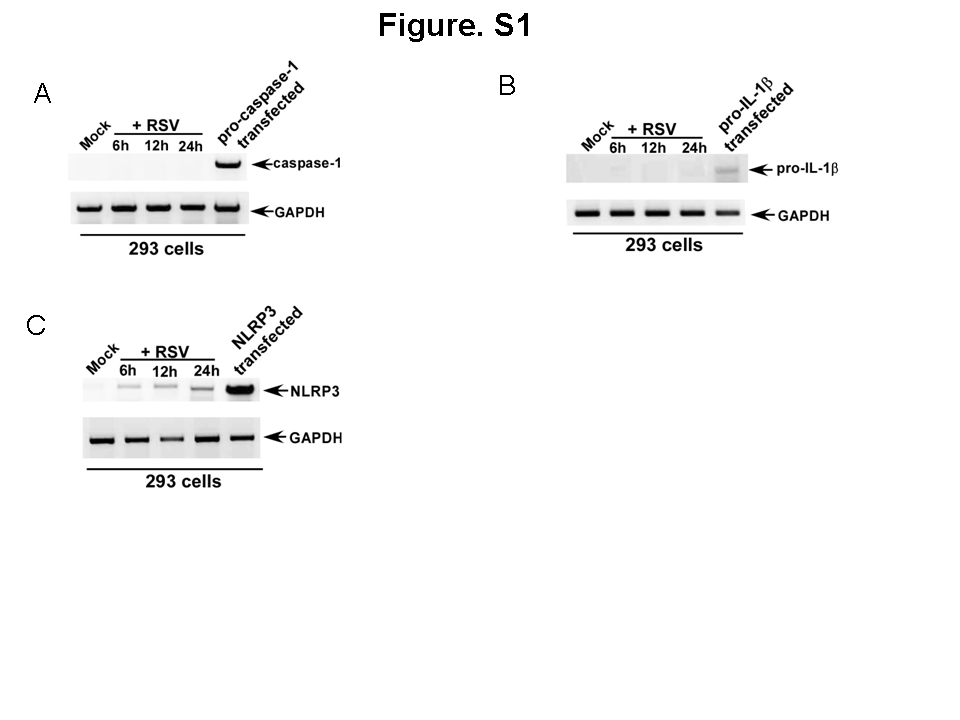

Supplement: Figure S1 — (A) RT-PCR analysis of caspase-1 expression in mock and RSV infected 293 cells. Caspase-1 expression was also monitored in RSV infected (at 24 h post-infection) 293 cells transfected with pro-caspase-1 plasmid (pro-caspase-1 transfected). (B) RT-PCR analysis of pro-IL-1β expression in mock and RSV infected 293 cells. Pro-IL-1β expression was also monitored in RSV infected (at 24 h post-infection) 293 cells transfected with pro-IL-1β plasmid (pro-IL-1β transfected). (C) RT-PCR analysis of NLRP3 expression in mock and RSV infected 293 cells. NLRP3 expression was also monitored in RSV infected (at 24 h post-infection) 293 cells transfected with NLRP3 plasmid (NLRP3 transfected). (TIF) [file pone.0029695.s001.tif]

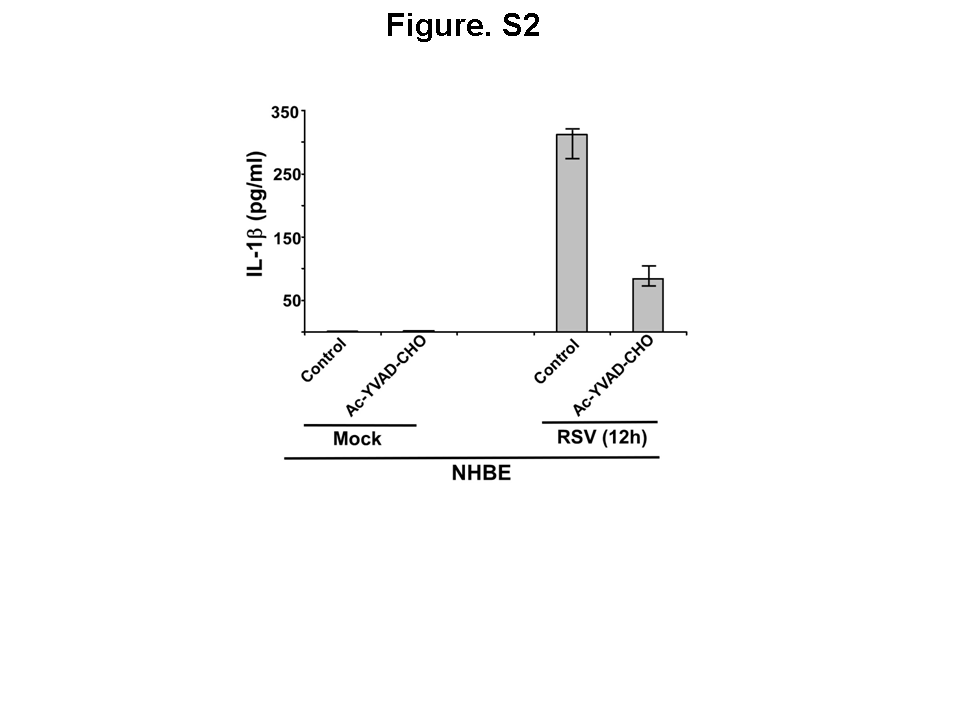

Supplement: Figure S2 — Primary normal human bronchial epithelial (NHBE) cells were infected with RSV (1 MOI) in the presence of either water (vehicle control) or caspase-1 inhibitor (10 µM of Ac-YVAD-CHO). IL-1β levels in the medium supernatant were assayed by ELISA at 12 h post-infection. Each value represents the mean ± standard deviation from three independent experiments. (TIF) [file pone.0029695.s002.tif]

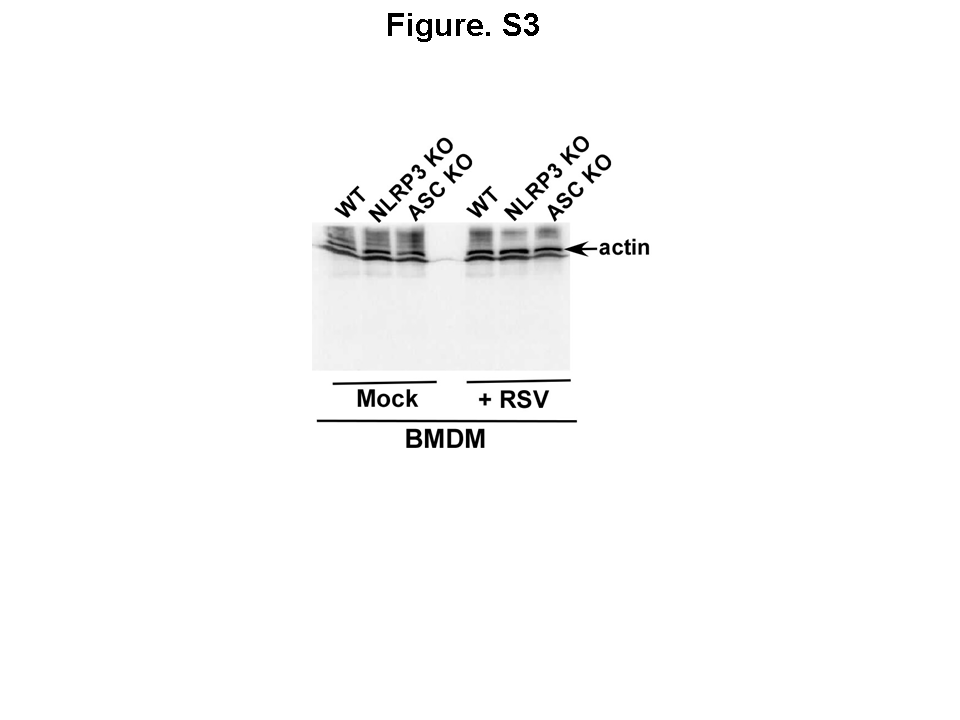

Supplement: Figure S3 — Wild type (WT), NLRP3 knock-out (KO) and ASC KO BMDMs were infected with RSV (1 MOI) for 12 h. The cell lysate from mock infected and RSV infected cells were subjected to Western blot analysis with mouse caspase-1 p10 subunit specific antibody (as shown in Fig. 4A). The blot was stripped and re-probed with anti-actin antibody. (TIF) [file pone.0029695.s003.tif]

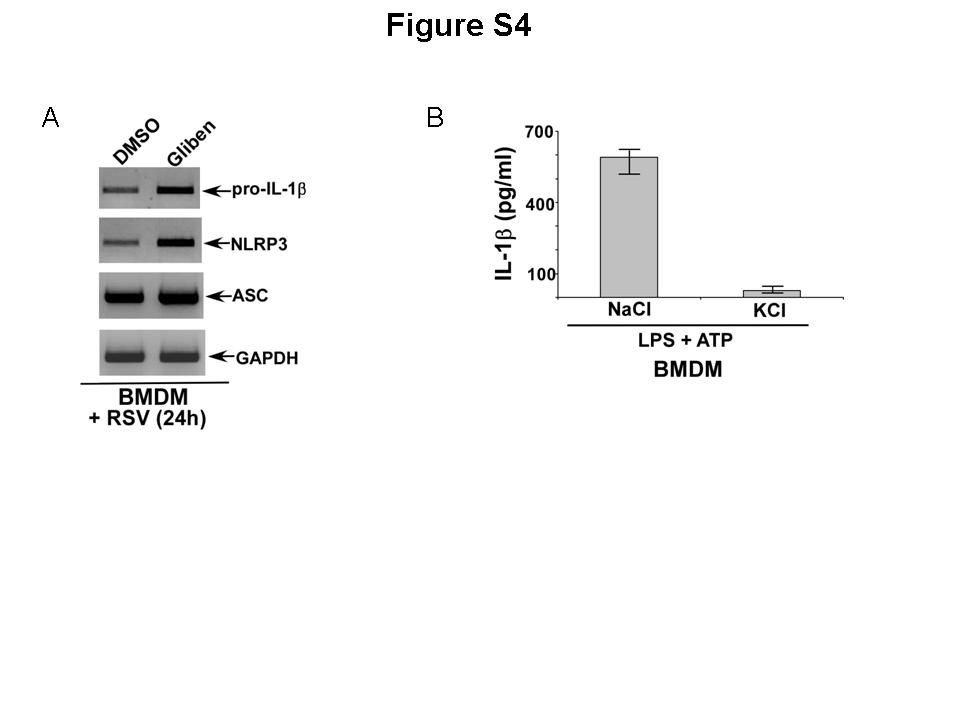

Supplement: Figure S4 — (A) RT-PCR analysis of pro-IL-1β, ASC, and NLRP3 expression in RSV infected wild type primary mouse bone marrow derived macrophages (BMDM) treated with either DMSO or glibenclamide (Gliben) (50 µM). (B) WT BMDMs were treated with LPS+ATP (primed with LPS for 12 h, followed by stimulation with ATP for 30 mins) in the presence of buffer containing either 150 mM NaCl (control) or 150 mM KCl. IL-1β levels in the medium supernatant were assayed by ELISA. Each value represents the mean ± standard deviation from three independent experiments. (TIF) [file pone.0029695.s004.tif]
